# Supplementary material for: Ni(II) Ions May Target the Entire Melatonin Biosynthesis Pathway—A Plausible Mechanism of Nickel Toxicity
Source: Molecules. 2022 Aug 30;27(17):5582. doi: 10.3390/molecules27175582 (PMC9458082; doi:10.3390/molecules27175582)
Supplement: Supplementary file 1 [file molecules-27-05582-s001.zip › molecules-1889008-supplementary.pdf]

Supplementary Information

Ni(II) Ions May Target the Entire Melatonin Biosynthesis Pathway — a Plausible Mechanism of Nickel Toxicity

Nina E. Wezynfeld [1,2], Arkadiusz M. Bonna [1,3], Dawid Płonka [1], Wojciech Bal [1] and Tomasz Frączyk [1,*]

[1] Institute of Biochemistry and Biophysics, Polish Academy of Sciences, 02-106 Warsaw, Poland
[2] Faculty of Chemistry, Warsaw University of Technology, 00-664 Warsaw, Poland
[3] Department of Biochemistry, University of Cambridge, Cambridge CB2 1QW, United Kingdom
* Correspondence: tfraczyk@ibb.waw.pl

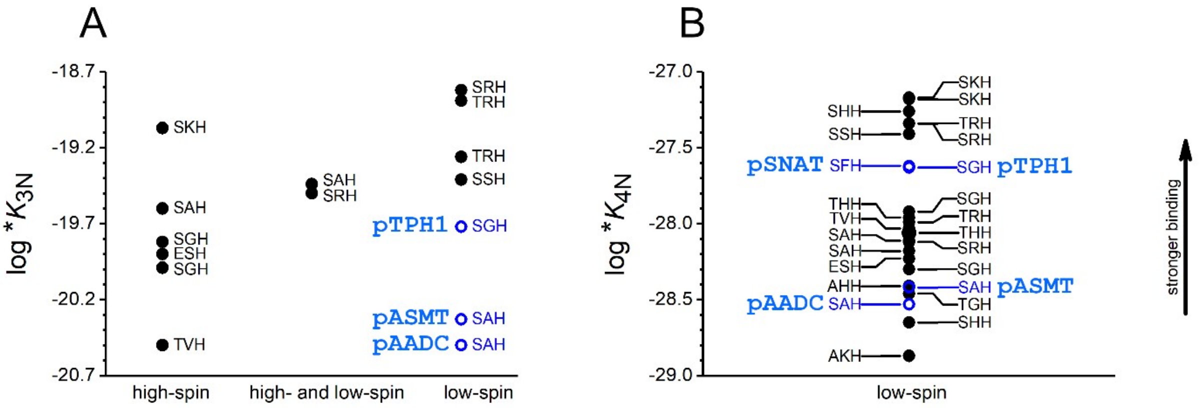

Figure S1. Protonation-corrected logarithmic stability constants of Ni(II) 3N (A, log $^*K_{3N}$), and 4N (B, log $^*K_{4N}$) complexes and spin states of these complexes. The considered peptides are those derived from the melatonin biosynthesis pathway marked by blue open circles, Ac-HALSGHAKV-am (SGH, pTPH1); Ac-TVESAHVQR-am (SAH, pAADC); Ac-ERFSFHAVG-am (SFH, pSNAT); Ac-VRASAHGTE-am (SAH, pASMT); and other, previously studied peptides marked by black dots, Ac-TKYSKHDMN-am (SKH); Ac-GVGTRHKAL-am (TRH); Ac-KMSTVHEIL-am (TVH); Ac-SALSGHLET-am (SGH); Ac-KALTGHLEE-am (TGH); Ac-VKSSSHFNP-am (SSH); Ac-GASGHAKFL-am (SGH); Ac-GASAHWKFL-am (SAH); Ac-GASKHWKFL-am (SKH); Ac-GASRHAKFL-am (SRH); Ac-GASRHWKFL-am (SRH); Ac-GATRHWKFL-am (TRH); Ac-GATHHWKFL-am (THH); Ac-TESHHK-am (SHH); Ac-TASHHK-am (SHH); Ac-TEAHHK-am (AHH); Ac-TESAHK-am (SAH); Ac-TESHAK-am (ESH); Ac-TETHHK-am (THH); Ac-ELAKHA-am (AKH) [1–5].

References

1. Wezynfeld, N.E.; Bossak, K.; Goch, W.; Bonna, A.; Bal, W.; Frączyk, T. Human Annexins A1, A2, and A8 as Potential Molecular Targets for Ni(II) Ions. *Chem. Res. Toxicol.* **2014**, *27*, 1996–2009, doi:10.1021/tx500337w.
2. Kopera, E.; Krężel, A.; Protas, A.M.; Belczyk, A.; Bonna, A.; Wysłouch-Cieszyńska, A.; Poznański, J.; Bal, W. Sequence-Specific Ni(II)-Dependent Peptide Bond Hydrolysis for Protein Engineering: Reaction Conditions and Molecular Mechanism. *Inorg. Chem.* **2010**, *49*, 6636–6645, doi:10.1021/ic1005709.
3. Mylonas, M.; Krężel, A.; Plakatouras, J.C.; Hadjiliadis, N.; Bal, W. The Binding of Ni(II) Ions to Terminally Blocked Hexapeptides Derived from the Metal Binding -ESHH- Motif of Histone H2A. *J. Chem. Soc., Dalton Trans.* **2002**, 4296–4306, doi:10.1039/B206585A.
4. Krezel, A.; Mylonas, M.; Kopera, E.; Bal, W. Sequence-Specific Ni(II)-Dependent Peptide Bond Hydrolysis in a Peptide Containing Threonine and Histidine Residues. *Acta Biochim. Pol.* **2006**, *53*, 721–727.
5. Karavelas, T.; Malandrinos, G.; Hadjiliadis, N.; Mlynarz, P.; Kozlowski, H.; Barsam, M.; Butler, I. Coordination Properties of Cu(II) and Ni(II) Ions towards the C-Terminal Peptide Fragment -TYTEHA- of Histone H4. *Dalton Trans.* **2008**, 1215–1223, doi:10.1039/B716863B.
